# Experience of using beta-D-glucan assays in the intensive care unit

**DOI:** 10.1186/s13054-018-2044-7

**Published:** 2018-05-12

**Authors:** A. Dagens, N. Mughal, A. Sisson, L. S. P. Moore

**Affiliations:** 1 Chelsea and Westminster Hospital, 369 Fulham Rd, Chelsea, London, SW10 9NH UK; 2 Royal Air Force, London, UK; 3 NWL Pathology at Imperial College Healthcare NHS Trust, Fulham Palace Road, London, W6 8RF UK; 4 Imperial College London, Exhibition Road, London, UK

We read with interest the article by Giacobbe et al. [1] assessing the combined performance of serum (1,3)-β-D-glucan (BDG) and procalcitonin (PCT) for the differential diagnosis of candidaemia and bacteraemia in the intensive care setting.

It is our experience that some intensivists lack familiarity with the role of BDG assays and that it is often not a readily available test. To understand how BDG assays were being used in our department we undertook a retrospective observational study. Ours is a central London teaching hospital with a 13-bed mixed medical/surgical ICU. All BDG assays sent from the ICU to a national reference laboratory over a 3-year period (September 2014 to March 2017) were extracted, and clinical and laboratory parameters collated to assess the impact on patient outcomes.

Thirty assays were identified among 704 patients, with a mean patient age of 58 (range 21–86). Most (11/30) of these patients had been admitted to ICU for respiratory disease. A clinical candida score was calculated for each patient (mean 3, mode 4), suggesting a high pre-test probability for invasive fungal disease (IFD).

The average turnaround time was 5 days (range 3–10 days), with 6/30 positive. Twenty-six patients had been started on antifungal medication empirically whilst awaiting assay results. On return of negative assay results five patients had their antifungals stopped within 24 h of the result; 120 patient-days of antifungal treatment were given whilst awaiting negative assay results.

Serological BDG assays are a potentially useful tool in the diagnosis of IFD and can facilitate antifungal stewardship. However, complicating factors limit their use, particularly where outsourcing to an external laboratory prolongs turnaround times. Antifungal agents are expensive. Based upon NHS list prices for our formulary echinocandin, this study identified £49,920 spent unnecessarily on antifungal agents.

Mortality was high in our cohort (50%), perhaps reflecting the fact that IFD is often only considered in patients who are not improving in ICU. It was noticeable how few assays were sent during the 3-year period, possibly reflecting a lack of awareness of BDG amongst intensivists.
